# Persistent Memory in Single Node Delay-Coupled Reservoir Computing

**DOI:** 10.1371/journal.pone.0165170

**Published:** 2016-10-26

**Authors:** André David Kovac, Maximilian Koall, Gordon Pipa, Hazem Toutounji

**Affiliations:** 1 Neuroinformatics Department, Institute of Cognitive Science, University of Osnabrück, Osnabrück, Lower Saxony, Germany; 2 Department of Theoretical Neuroscience, Central Institute of Mental Health, Medical Faculty Mannheim of Heidelberg University, Mannheim, Baden-Württemberg, Germany; University of Sheffield, UNITED KINGDOM

## Abstract

Delays are ubiquitous in biological systems, ranging from genetic regulatory networks and synaptic conductances, to predator/pray population interactions. The evidence is mounting, not only to the presence of delays as physical constraints in signal propagation speed, but also to their functional role in providing dynamical diversity to the systems that comprise them. The latter observation in biological systems inspired the recent development of a computational architecture that harnesses this dynamical diversity, by delay-coupling a single nonlinear element to itself. This architecture is a particular realization of Reservoir Computing, where stimuli are injected into the system in time rather than in space as is the case with classical recurrent neural network realizations. This architecture also exhibits an internal memory which fades in time, an important prerequisite to the functioning of any reservoir computing device. However, fading memory is also a limitation to any computation that requires persistent storage. In order to overcome this limitation, the current work introduces an extended version to the single node Delay-Coupled Reservoir, that is based on trained linear feedback. We show by numerical simulations that adding task-specific linear feedback to the single node Delay-Coupled Reservoir extends the class of solvable tasks to those that require nonfading memory. We demonstrate, through several case studies, the ability of the extended system to carry out complex nonlinear computations that depend on past information, whereas the computational power of the system with fading memory alone quickly deteriorates. Our findings provide the theoretical basis for future physical realizations of a biologically-inspired ultrafast computing device with extended functionality.

## Introduction

Some neuron types are endowed with extensive dendritic trees. Each dendrite is characterized by its spatial location within the tree, and the *delay* required for a postsynaptic action potential to propagate to the soma. While several studies investigate the computational role of the dendrites’ spatial distribution [1–3], the functionality of dendritic propagation delays is scarcely probed. One suggestion is that propagation delays enrich the dynamics of recurrent neural networks by turning them into *infinite-dimensional dynamical systems* [4]. The latter observation was the basis of a neurally-inspired computational paradigm, the *single node Delay-Coupled Reservoir* (DCR), where a single nonlinear neuron is delay-coupled to itself [5, 6]. Inputs are multiplexed in time across the delay, and are nonlinearly processed at the neural site. The DCR provides a promising testing bed to theories of neural computations with delays. For instance, some of the authors have shown that applying homeostatic plasticity [7] directly to the delays dramatically improved the computational capabilities of the DCR [8].

In this article, we demonstrate that adding a trained feedback, or *teacher forcing*, to the DCR endow the latter with the ability to store stable memories. The DCR is a specific realization of *Reservoir Computing* (RC) [9–12], which is a flexible framework for capturing temporal dependencies in time series of complex natural systems. This ability is a necessary ingredient in neural information processing [13–16], in addition to spawning a wide range of applications, including time series forecasting [17], signal generation [18] and robot navigation [19].

RC models are large, driven, nonlinear dynamical systems, such as a recurrent neural network, which map their input to a high-dimensional space. On the one hand, the recurrency allows input to travel within the dynamical system, or *reservoir*, for a certain period of time, resulting in a form of *short-term memory*. On the other hand, random nonlinear motifs within the reservoir *nonlinearly* mix past and present inputs. Together, memory and the nonlinear mixing allow a desired output to be *linearly* combined from the activity of the reservoir by output units, using linear regression.

Teacher forcing is a technique originally used in training recurrent neural networks to approximate trajectories of dynamical systems [20, 21]. Output units are clamped to their target value during training, which assures a low amount of training error that, otherwise, would limit the neural network’s ability to learn target trajectories. The same principle was also applied successfully to RC with sigmoidal recurrent neural networks and has shown to improve the ability of predicting chaotic trajectories by several orders of magnitude [17]. An alternative training mechanism avoids large training error and the resulting instability of learning by enforcing a rapid decrease in output error at early stages of learning [22, 23]. This procedure, called FORCE, requires chaotic dynamics in the spontaneous activity of the recurrent neural network. The current paper, however, only considers fixed point spontaneous DCR dynamics in concord with previous studies [5, 8, 24], and exploring a setup similar to FORCE will be carried out elsewhere. In addition, in its original form, a DCR only retains temporal dependencies on a short time scale. The goal of the current paper is to extend the DCR’s applicability to cases that require *stable memories*, while enhancing its ability to encode and predict dynamical systems by teacher forcing or FORCE is beyond its scope. In classical RC based on sigmoidal and spiking recurrent neural networks, stable memories were shown to be realizable by the aid of teacher forcing and dedicated output units [25, 26], which is the approach we follow to endow the DCR with this capability.

As in classical RC, a DCR receives an input stream that perturbs its autonomous dynamics. Instead of the spatial distribution of input across many neurons, computation in the DCR is implemented by using *time-multiplexing* at several dendritic arbors across the delay line [27]. This leads to *virtual nodes*, at which the dynamics of the single delay-coupled neuron is sampled. Despite these differences, a DCR is approximately equivalent to a recurrent neural network with constrained connectivity [5, 6, 8]. This translates to comparable performance [5], as well as similar principles of self-organization based on plasticity [28–30] that can improve the DCR in an unsupervised fashion [8]. At the same time, the simple architecture of the DCR allows for a largely reduced complexity in physical implementation, which has already been demonstrated on electronic [5], optoelectronic [31, 32] (with the input either in synchrony with [31] or asynchronous to [32] the delay line), and ultrafast all-optical hardware [33]. Hence, DCRs have the potential for dramatic changes in the field of biologically-inspired ultrafast computation, based on new physical realizations, which is reflected in the fast growing attention paid to this field of research [6].

A standard RC architecture, including the DCR, undergoes rapid washout of previous inputs, a property that is termed *fading memory* [34]. This property assures the execution of computations that demand input retrieval for several time steps in the immediate past. However, systems with fading memory fail at computations that require stable storage of relevant features for arbitrary length of time. Luckily, as pointed out above, this limitation can be overcome by teacher forcing [25]. The latter leads to a stabilization of a finite number of memorized states, and therefore extends the class of executable computations. Here, we demonstrate that such feedback can also be employed to stabilize memory in DCRs. Based on simulations, we show that DCRs which incorporate trained feedback are able to have memory traces of an arbitrary length.

The article is structured as follows. We start with describing the RC architecture that is based on a single nonlinear node with delayed feedback, i.e., the DCR. We then present how the DCR can be extended by linear feedback. This is followed by numerical simulations, which demonstrate the role of teacher forcing in stabilizing memory, while preserving the system’s ability to perform nonlinear computations. These simulations consist of three experiments, showing that the enhanced system is able to learn complex nonlinear tasks requiring long-term memory that cannot be learned by classical DCRs. At last, we demonstrate that this memory can be maintained for practically infinite time.

## Materials and Methods

### Delay-based Reservoir Computing

**Fig 1:**
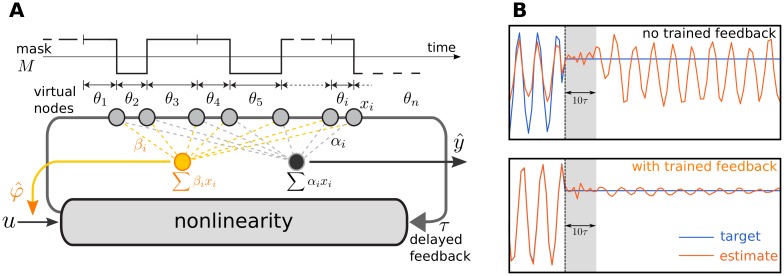
DCR with trained feedback (fDCR) and its extended functionality. (A) In a fDCR, the input *u* and trained feedback signals *φ* are *temporally multiplexed* across a delay line of length *τ*, by using random binary masks *M* of *n* bits each (only one mask shown). Each mask bit *M*_*i*_ is held constant for a short delay *θ*_*i*_, such that ∑*θ*_*i*_ = *τ*. The masked input is then nonlinearly transformed and mixed with past input by the nonlinear node with delayed feedback. At the end of each *θ*_*i*_, resides virtual nodes with activity *x*_*i*_. Feedback coefficients *β*_*i*_ are estimated through *teacher forcing* to allow storing stable memories, and feedforward coefficients *α*_*i*_ are then trained to perform computations through linear regression. (B) With no trained feedback (top), the DCR is not capable of retaining cue information (dashed vertical line) beyond the bounds of fading memory (∼10*τ*). With trained feedback (bottom), memory of the cue is stabilized, allowing the fDCR’s output (orange) to track the change in the target function (blue) beyond the bounds of fading memory.

In a DCR, the recurrent neural network in classical RC is replaced by a single nonlinear node with delayed feedback. Past and present inputs $u\in R^{m}$ undergo nonlinear mixing via injection into the nonlinear node. Formally, the dynamics can be modeled by a forced (or driven) *Delay Differential Equation* (DDE) of the form

$$\dot{x}\left(t\right)=-x\left(t\right)+f\left(x(t-\tau ),u(t)\right),$$ (1)

where *τ* is the delay time, and x(t),x(t-τ)∈R are the current and delayed DCR activities. Fig 1 illustrates the full DCR setup with trained feedback.

DCRs were successfully implemented both virtually and physically. Despite variable performance of different implementations, the principal computational properties remain invariant. Here, we restrict our simulations to the Mackey-Glass system [35]. This choice of nonlinearity is motivated by its superior performance, and the possibility of approximating it by electronic circuits [5]. After a proper transformation *M* of the input (see below), the input-driven Mackey-Glass DCR is modeled by:

$$\dot{x}\left(t\right)=-x\left(t\right)+\frac{\eta \left(x(t-\tau )+\gamma Mu(t)\right)}{1+{\left(x(t-\tau )+\gamma Mu(t)\right)}^{\rho }}$$ (2)

where *γ*, *η* and *ρ* are model parameters, the latter regulating the chaoticity of the system.

Solving the system (1) for *t* ≥ 0 requires specifying an appropriate *initial value function* $\phi_{0}:\left[-\tau ,0\right]\to R$. This suggests that the phase space in which the solution resides is a *Banach space* $C_{1,\tau }=C([-\tau ,0],R)$ which is *infinite dimensional* [36]. This entails that using a DDE as a reservoir provides the high-dimensional expansion of input, usually achieved by using a large number of neurons.

Instead of distributing an *m*-dimensional input spatially across neurons, input to the DCR is *time-multiplexed*, which is carried out as follows:

The DCR receives a constant input $u\left(\bar{t}\right)\in R^{m}$ in each reservoir time step $\bar{t}=\left\lceil \frac{t}{\tau }\right\rceil$, corresponding to one *τ*-cycle of the system. The input is then linearly transformed by a mask *M* that is piecewise constant for short periods *θ*_*i*_. These represent the delays between sampling points of *i* = 1, …, *n* *virtual nodes* along the delay line. Accordingly, the delays between the virtual nodes satisfy $\sum_{i=1}^{n}\theta_{i}=\tau$, where *n* ≫ *m* is the effective dimensionality of the DCR. Here, the mask *M* is binary with random mask bits *M*_*i*_ ∈ {−*μ*, +*μ*}^*m*^, so that the virtual node *i* receives a weighted input $M_{i}u\left(\bar{t}\right)$. In order to assure that the DCR possesses fading memory of the input, the system (1) is set to operate, when unforced, in a single fixed point regime. Thus, the masking procedure effectively prevents the driven dynamics of the underlying system from saturating to the fixed point.

Following the time-multiplexing of input, a sample of the DCR’s response is read out at the end of each *θ*_*i*_. This yields *n* predictors *x*_*i*_ per time step $\bar{t}$, corresponding to the virtual nodes’ activity. Computations are performed on the predictors using a linear regression model for some scalar target time series *y*, given by $\hat{y}\left(\bar{t}\right)=\sum_{i=1}^{n}\alpha_{i}x_{i}\left(\bar{t}\right)$. The coefficients *α*_*i*_ are determined by using the *least squares solution*, minimizing the sum of squared errors $\sum_{\bar{t}}{\left(y\left(\bar{t}\right)-\hat{y}\left(\bar{t}\right)\right)}^{2}$. These linear readouts are called *feedforward readouts* to distinguish them from *feedback readouts* of the extended DCR.

### Feedback readouts for stabilizing memory

In an RC architecture, parameters need to be tuned such that it possesses fading memory. This is achieved in DCRs by setting the nonlinearity to operate in a fixed point regime, in addition to masking the input as outlined above. Similar to classical RC, possessing fading memory alone restricts the class of computations a DCR can carry out to those that depend on relatively recent inputs only. In order to overcome this restriction, we rely on an important theoretical result for conventional RC [25]. This result states that under certain conditions, augmenting the system with trained feedback allows it to store nonfading memory. The same extension can be applied to the DCR, leading to similar boost in its computational capability.

More precisely, input is extended by additional channels $\varphi \left(\bar{t}\right)\in R^{q}$, which are the fed back outputs $\hat{z}\left(\bar{t}-1\right)=\sum_{i=1}^{n}\beta_{i}x_{i}\left(\bar{t}-1\right)$ at the previous reservoir time step $\bar{t}-1$ of a subset of linear readouts. The resulting DCR with trained feedback (fDCR) is shown in Fig 1. The regression coefficients *β*_*i*_ of these feedback readouts are estimated offline at the end of initial teacher forcing phase that precedes the training of feedforward readouts: The reservoir is fed with training data $\left(u\left(\bar{t}\right),\tilde{\varphi }\left(\bar{t}\right)\right)\in R^{m+q}$, where training feedback signals are replaced by a noisy version of their target values $\tilde{\varphi }\left(\bar{t}\right)=\tilde{z}\left(\bar{t}-1\right)=z\left(\bar{t}-1\right)+\epsilon \left(\bar{t}-1\right)$. Adding noise *ϵ* assures that at later phases, the feedback readouts are robust to noise, i.e., prediction errors in the trained feedback are not amplified due to overfitting [25]. The feedback coefficients *β*_*i*_ are determined by using the least squares solution, minimizing the sum of squared errors $\sum_{\bar{t}}{\left(\tilde{z}\left(\bar{t}\right)-\hat{z}\left(\bar{t}\right)\right)}^{2}$.

**Fig 2:**
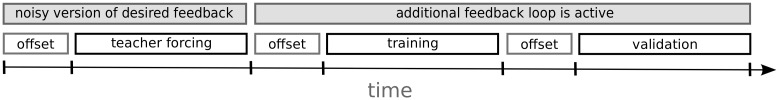
Training and validation of fDCR. Input-output pairs are split into three consecutive phases. In the *teacher forcing* phase, the feedback readouts are trained. In the *training phase*, feedforward readouts are trained to solve computational tasks that require both fading and nonfading memory. in the *validation phase*, the model performance is assessed with unseen data. Each phase is preceded by a brief offset for the fading memory of the fDCR to wash out.

Following teacher forcing, feedforward coefficients *α*_*i*_ are estimated offline at the end of the training phase, outlined in the previous section. The model is then validated on new input and feedback time series. Feedback signals in both training and validation phases are computed by $\hat{\varphi }\left(\bar{t}\right)=\sum_{i=1}^{n}\beta_{i}x_{i}\left(\bar{t}-1\right)$. The full procedure is shown in Fig 2.

## Computational tasks

In order to examine the ability of the fDCR to retain memory traces for time spans which exceed fading memory, we designed three tasks whose proper execution requires the presence of long-term memory. The exact experimental setups and typical results are presented in the Results section.

In the first two tasks the target function switches between two computations on one or more input streams where each of these computations on its own does not require long-term memory. The switch between the two distinct computations is triggered by short cues, which are received alternately via two additional input streams. A cue in the first cue channel triggers a switch from one task to the other, while the second cue triggers the opposite switch. In order to learn the described tasks, the fDCR has to preserve a memory trace of the last cue at every point in time. This is an easy task if the duration between cues is within the bounds of fading memory, i.e., smaller than around 10 reservoir time steps. If, instead, time gaps between two successive cue signals exceed this limit, the resulting long-term memory dependent task cannot be learned by the standard DCR. This in mind, experiments here are designed with gaps between cues which exceed the fading memory trace by at least tenfold. In order to control for the possibility that feedback readouts are only learning to generate periodic signals, independent of cue time, cues are irregularly spaced.

In the third experiment, more complex feedback signals are trained to encode the time since last cue. This task is designed to demonstrate that the fDCR is not only capable of registering the binary information of a cue’s presence or absence, but also the time since the last cue has been shown, and to use this information in computation. This information is stored in the value of a ramping feedback signal. The time scale at which time is stored is defined by slope of the ramp length. Longer ramps corresponds to higher sensitivity to the time of older cues.

We follow these three experiments with a longitudinal simulation, that serves to demonstrate how stable cue storage is.

### Model parameters

Mackey-Glass parameters as they appear in Eq (2) in addition to all other fDCR parameters are fixed across all simulations, and are summarized in Table 1.

**Table 1:** Parameter values of the Mackey-Glass system and the fDCR.

Parameter	Value	Description
*γ*	0.01	scaling factor of input
*η*	0.5	scaling factor of delayed nonlinearity
*ρ*	1^a^	regulating parameter of chaoticity
±*μ*	±0.1	values of mask bits
*n*	300	number of virtual nodes
*τ*	600	delay time

The first group of parameters corresponds to the parameters of the Mackey-Glass system 2, while the second corresponds to the parameters of the fDCR.

^a^This choice of *ρ* sets the unforced Mackey-Glass system to operate in a single fixed point regime.

Consecutive cue onsets are separated by gaps that are uniformly drawn from the ranges [50, 400] and [100, 800] reservoir time steps for teacher forcing and for training and validation, respectively. The gaps during teacher forcing are shorter to assure that the regression sees more cues. Otherwise, the sparsity of cues would lead the regression for feedback weights to minimize square errors by assuming that no cues exist. Cues have a duration of 5 reservoir time steps.

### Simulation

The DDE Eq 1 was numerically solved using the recursive *method of steps* for handling delays, and *Heun’s method* for numerical integration. Heun’s method assures quadratic decay of errors with respect to discretization time step. The numerical solution is evaluated at 600 simulation points across the overall delay *τ*. The latter contains 300 virtual nodes, distributed randomly over the simulation points, such that ∑_*i*_ *θ*_*i*_ = *τ*.

Each experiment follows the training and testing procedure outlined in Fig 2. Table 2 shows the number of reservoir time steps in each simulation phase.

**Table 2:** Number of reservoir time steps within each phase of simulation.

Phase	Duration (in reservoir time steps)	
	Experiments 1 and 2	Experiment 3
offset	50	50
teacher forcing	50000	50000
training	10000	20000
validation	10000	20000

## Results

### Experiment 1: Switching between a sine function and a constant value

**Fig 3:**
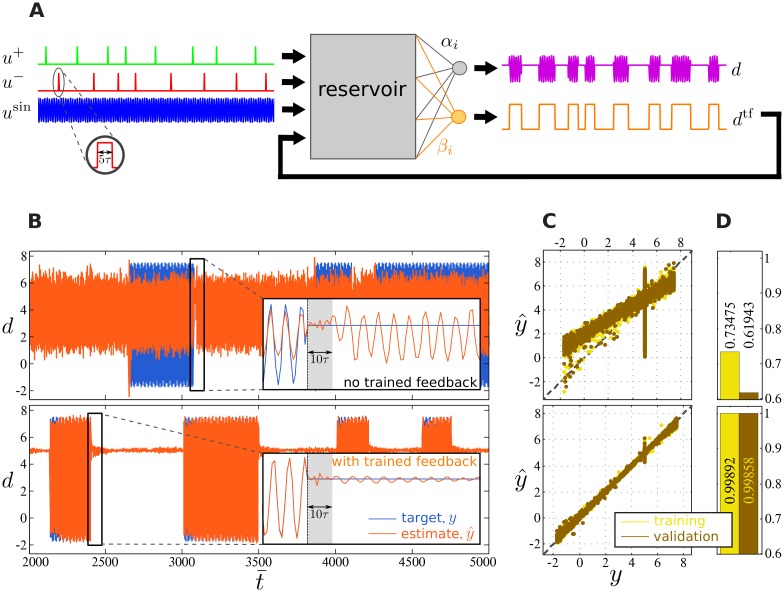
Experiment 1: Switching between a sine wave and the constant value 5. (A) Schematic of the experiment. Input consists of four streams: two cue channels *u*^+^ and *u*^−^, a sine wave *u*^sin^, and the additional trained feedback signal *d*^tf^. First, feedback weights (orange) are learned by teacher forcing, followed by the feedforward weights (gray). Successful learning is achieved when the output signal *d* (magenta) matches its target value. (B) Comparison between the output signal (orange) and target value (blue) with (bottom) and without (top) trained feedback. (C) Scatter plots of the target signal *y* verses observed output $\hat{y}$ with (bottom) and without (top) trained feedback, and (D) their correlation coefficient for training (brown) and validation data sets (yellow).

The setup of the fDCR for this experiment is depicted in Fig 3A. The target output *d* is either the oscillatory *u*^sin^ (a sine wave $100\sin(\bar{t})+1/3$, filtered by a Gaussian kernel with *std* = 5*τ*) after the cue onset in the input channel *u*^+^, or is constant at 5 after cue onset in input channel *u*^−^. The trained feedback signal *d*^tf^ represents the fourth input stream.

A closer look at the output signal immediately following cue *u*^−^ onset in the top panel of Fig 3B demonstrates that the standard DCR produces output that is fairly close to the desired constant value. However, only after a few reservoir time steps the cue’s fading memory vanishes, and the input-driven system returns to oscillate in synchrony with its input. Thus, the desired signal could not be retained over the entire time span between two consecutive cues. This instability is due to the fact that the readout neuron has to transform transient information (caused by permanent input) into a stable output. Yet, approximating constant output is very difficult for reservoir computing [10]. The mismatch between the desired and observed output signals is also demonstrated by the divergence from diagonal of their corresponding scatter plots (upper panel in Fig 3C), particularly when the desired output is at the constant value.

The fDCR, on the other hand, overcomes this limitation, as shown in the bottom panel of Fig 3B. The close match between desired and observed signals demonstrates that trained feedback is successfully utilized in order to stabilize the memory of the last cue (also see lower panel in Fig 3C). We also note that the fDCR approximates the desired output with increasing accuracy over time.

Finally, to quantify these observations, we compute correlation coefficients between the desired and observed signals for both training and validation sets (Fig 3D). We particularly note that, in addition to significantly higher correlation in the case of fDCR, the correlations for training and validation data are very similar (see lower panel in Fig 3D). This demonstrates that the fDCR generalizes well and better than standard DCR (upper panel in Fig 3D).

### Experiment 2: Concurrent linear and nonlinear tasks

We demonstrated that including trained feedback stabilizes memories of cue signals. We now show that the computational resources of the fDCR are not fully depleted by the demands of learning these signals. Mainly, we show that the fDCR is still capable of performing several (potentially nonlinear) computations concurrently, some of which are cue-independent.

**Fig 4:**
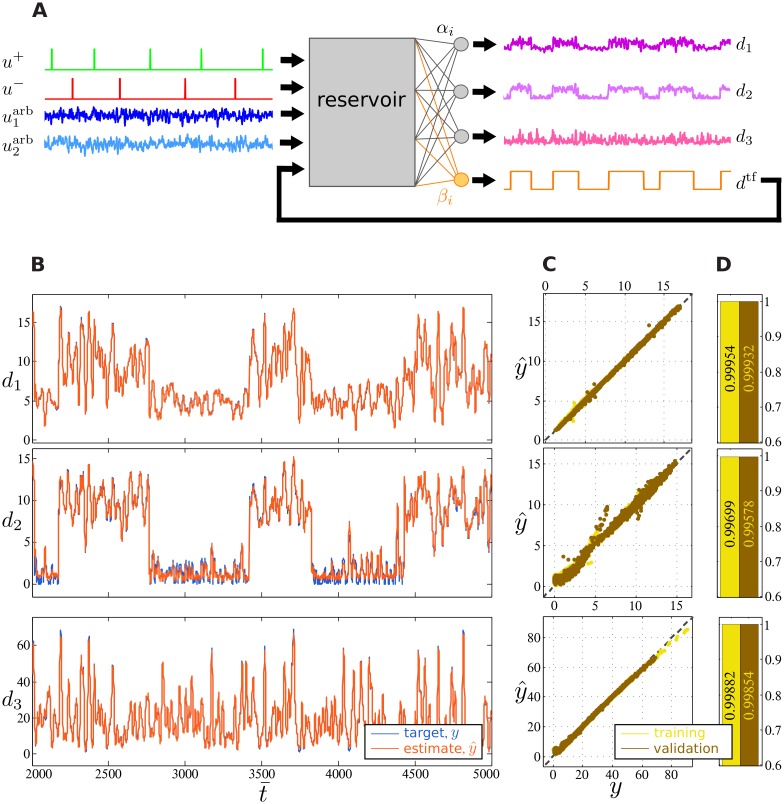
Experiment 2: Concurrent linear and nonlinear tasks. (A) Schematic of the experiment. Input consists of five streams: two cue channels *u*^+^ and *u*^−^, two streams of bounded, uniformly distributed noise $u_{1}^{\text{arb}}$ and $u_{2}^{\text{arb}}$ that are filtered by a Gaussian kernel, and the additional trained feedback signal *d*^tf^. In addition to the trained feedback signal, the fDCR computes three desired outputs *d*_1_, *d*_2_, and *d*_3_, corresponding to a linear cued task, nonlinear cued task, and nonlinear cue-independent task, respectively. (B) Comparison between desired (blue) and observed (orange) fDCR output signal *d*_1_ (top), *d*_2_ (middle), and *d*_3_ (bottom). (C) Scatter plots of the target verses observed output for both training (yellow) and validation (brown) data sets, when the target is *d*_1_ (top), *d*_2_ (middle), and *d*_3_ (bottom). (D) Correlation coefficient between desired and observed fDCR outputs for both training (brown) and validation (yellow), when the target is *d*_1_ (top), *d*_2_ (middle), and *d*_3_ (bottom).

Experiment 2 is designed with this goal in mind, as shown in Fig 4A. Input to the fDCR consists of two cue channels *u*^+^ and *u*^−^, two streams of bounded and filtered uniformly distributed noise $u_{1}^{\text{arb}}$ and $u_{2}^{\text{arb}}$, and the additional trained feedback signal *d*^tf^. Input signals $u_{1}^{\text{arb}}$ and $u_{2}^{\text{arb}}$ are filtered with a Gaussian kernel (std = 5*τ*) to improve performance after being drawn uniformly from the range [−5, 15]. Nevertheless, the fDCR is still capable of learning computations on uniform white noise as well. The fDCR is trained to perform three computations $d_{i}\left(u_{1}^{\text{arb}},u_{2}^{\text{arb}}\right)$ for *i* = 1, 2, 3. These computations are given by:

$$d_{1}\left(\bar{t}\right)=\left\{\begin{array}{cc} u_{1}^{\text{arb}}\left(\bar{t}\right) & \;\text{when}\;u^{+}\\ 2u_{2}^{\text{arb}}\left(\bar{t}\right) & \;\text{when}\;u^{-} \end{array}\right.$$ (3)

$$d_{2}\left(\bar{t}\right)=\left\{\begin{array}{cc} u_{1}^{\text{arb}}\left(\bar{t}\right)+u_{2}^{\text{arb}}\left(\bar{t}\right) & \;\text{when}\;u^{+}\\ |u_{1}^{\text{arb}}\left(\bar{t}\right)-u_{2}^{\text{arb}}\left(\bar{t}\right)| & \;\text{when}\;u^{-} \end{array}\right.$$ (4)

$$d_{3}\left(\bar{t}\right)=0.1{\left[u_{1}^{\text{arb}}\left(\bar{t}\right)\right]}^{3}+0.2u_{1}^{\text{arb}}\left(\bar{t}\right)\cdot u_{2}^{\text{arb}}\left(\bar{t}\right)$$ (5)

The first computation *d*_1_ (Eq 3) is cued and linear. The second computation *d*_2_ (Eq 4) is also cued but is nonlinear, due to the absolute value computation upon the onset of *u*^−^. These two computations are performed concurrently using the same cue signals. The third computation *d*_3_ (Eq 5) is a nonlinear function of the two random input signals, and is independent of the cues.

As Fig 4B shows, feedforward linear readouts (orange) are able to closely track the desired signals. Only in the highly nonlinear *u*^−^-cued *d*_2_ computation, the scatter plot between the desired and observed signals diverges slightly from the diagonal (see middle panel in Fig 4C). This divergence results, however, in a minuscule reduction in correlation between the two signals, as shown in the middle panel of Fig 4D. The bottom panels of Fig 4B–4D show that, despite its independence from the trained feedback signal, the nonlinear *d*_3_ computation is performed with remarkable precision. This suggests that the fDCR dynamics is rich enough to support concurrent nonlinear computations, with no detectable interference between a trained feedback signal and those computations not dependent on it.

### Experiment 3: Feedback depending on time since last cue

Experiment 3 assesses different aspects which go beyond experiments 1 and 2. Here, the fDCR is simultaneously required to learn and maintain two feedback loops. Each feedback loop stores the time of last cue up to a certain threshold, one corresponding to fast $d_{f}^{\text{tf}}$ and the other to slow $d_{s}^{\text{tf}}$ forgetting of the time since last cue. This allows for computations that are not only a function of cue onset, but of its time, such as delayed response tasks.

**Fig 5:**
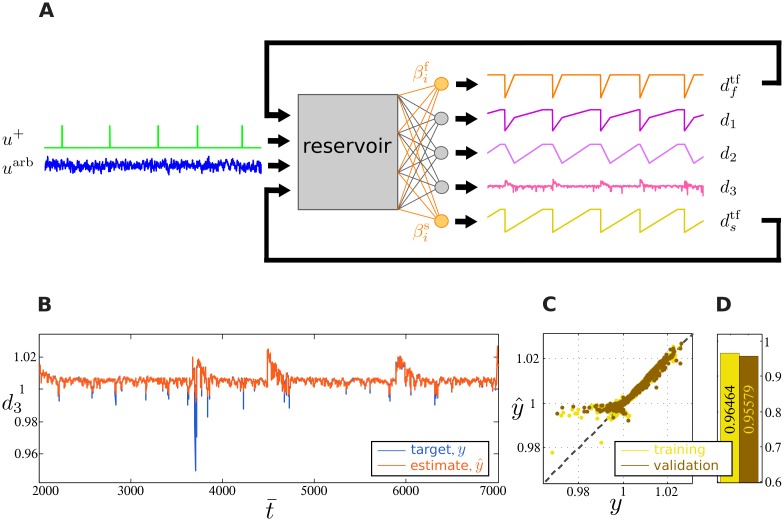
Experiment 3: Feedback depending on time since last cue. (A) Schematic of the experiment. Input consists of four streams: one cue channel *u*^+^, a stream of bounded, uniformly distributed noise *u*^arb^ that is filtered by a Gaussian kernel, and the additional fast $d_{f}^{\text{tf}}$ and slow $d_{s}^{\text{tf}}$ trained feedback signals that are ramps of duration 600 and 300 Reservoir time steps, respectively. In addition to the trained feedback signals, the fDCR computes three desired outputs *d*_1_, *d*_2_, and *d*_3_, corresponding to the sum of the feedback signals, their difference, and nonlinear function of the fast ramp and the random input, respectively. (B) Comparison between desired (blue) and observed (orange) fDCR output signal *d*_3_. (C) Scatter plots of the target verses observed output for both training (yellow) and validation (brown) data sets, when the target is *d*_3_. (D) Correlation coefficient between desired and observed fDCR output for both training (brown) and validation (yellow), when the target is *d*_3_.

This effect is implemented as follows. A cue triggers a sudden downward shift from amplitude 9 to 5 in each of the feedback signals. Instead of a sudden upward shift, feedback signals linearly increase back to a threshold value; i.e., a cue triggers a rising ramp. The two ramps are of different time scales, corresponding to 300 and 600 reservoir time steps for the short and a long ramp, respectively, as shown in Fig 5A. These time scales are larger than the fading memory capacity of the standard DCR in order to assess long-term stable memory of the time since last cue onset.

In contrast to the previous two experiments, the feedback signal itself is manipulated by cue onset in Experiment 3. Particularly, the target function *d*_3_ nonlinearly combines the random input stream *u*^arb^ with the fast ramp $d_{f}^{\text{tf}}$:

$$d_{3}\left(\bar{t}\right)={\left|u^{\text{arb}}\left(\bar{t}\right)\right|}^{-2.5\cdot d_{f}^{\text{tf}}\left(\bar{t}\right)}$$ (6)

As shown in Fig 5B shows, the output *d*_3_ is learned with high precision. A little mismatch occurs when the target signal *y* < 1, as the scatter plot Fig 5C demonstrates. However, the effect of this mismatch on the correlation between the target and desired signals is very little, as shown in Fig 5D, and does not result in overfitting the training data. In fact, the inability of the readout to track the target signal when *y* < 1 indicates that the readout mechanism is robust to outliers, since events where *y* < 1 are only sparsely present in the target time series (see S1 Fig). Further simulations demonstrate, as shown in S2 Fig, that the ability to carry out computations that depend on the time of cue onsets are not restricted to the function *d*_3_.

### Experiment 4: How stable is cue memory?

In order to answer this question, we ran a longitudinal simulation of a final experiment. It assesses how long the information of latest cue (as in Experiments 1 and 2) can be stored stably in the fDCR via the trained feedback loop.

The input to the fDCR for this experiment is similar to that of Experiment 1 (see Fig 3A), but with the sinusoidal input *u*^sin^ replaced by bounded random noise *u*^arb^ as in Experiment 2. This is to assure that long-term memory is robust to noise coming from that input channel.

Only one readout is trained by teacher forcing to generate the feedback signal. The ability of the fDCR is then tested for a number of time steps on generating the feedback signal in response to the two cues. Eventually, no cues were shown anymore in order to test how long the last cue may be maintained stably by the trained feedback. We term this phase of simulation the *stability test phase*. Simulation is canceled automatically if the feedback signal deviated beyond a certain error margin from its desired value.

**Fig 6:**
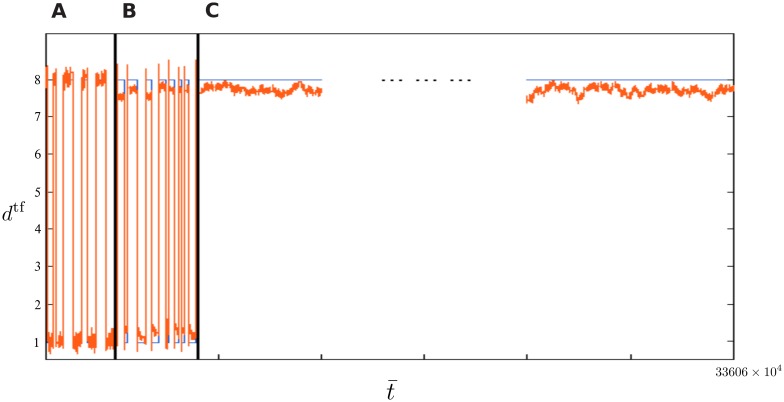
Experiment 4: stable storage of the cue through a longitudinal simulation. (A) A noisy version of the desired feedback signal (teacher forcing). (B) Trained feedback operating on input data with randomly timed cues. (C) Stability test phase where no more cues are input to the fDCR.

Fig 6 illustrates the results of this experiment. Following the teacher forcing and testing of trained feedback, the stability test was run for a day of physical time. Afterwards, simulation was interrupted manually, because the error margin was never exceeded. At this point, the fDCR has maintained its memory of the cue for ∼336*M* reservoir time steps. The feedback signal shows slight downward and upward shift due to modulation by the random input, but it never shows overall drift away from the desired value. Instead, the feedback signal seems to maintain a constant average value with no time limit.

## Discussion

As proven theoretically and confirmed through simulation by Maass and colleagues [25], trained feedback can overcome the limitations of fading memory in conventional reservoir computing. While the latter is modeled by a system of ordinary differential equations, here we show through simulation that this applies to single node Delay-Coupled Reservoirs, which are modeled by a single delay differential equation. The resulting fDCR (Fig 1A) successfully learns nonlinear long-term-memory-dependent tasks concurrently, and with high accuracy (Figs 3–5). We also show that memory storage is not only extended by an order of magnitude beyond fading memory, but is practically infinite (Fig 6). These simulations serve to demonstrate that with the added trained feedback, the fDCR combines sensitivity and stability. That is, the fDCR’s high-dimensional dynamics consists of transient input-sensitive representations, and attractor states where stable memories are stored.

A few issues remain to be addressed in the future. First, all tasks (Figs 3–5) require long-term memory and knowledge of the current input value only, with no demands for fading memory (in the exception of the feedback signal, which requires computing the highly-nonlinear exclusive-nor operation between the current and previous cue values [30]). The performance for more complex tasks, for which the target function depends on both long-term memory and fading memory, remain to be explored. We expect, as in the case of conventional reservoirs, that more complex computations require more complex systems. However, while complexity in conventional reservoirs can be controlled by the number of neurons, simply increasing the number of virtual nodes in a DCR does not immediately lead to improvement. This is because increasing the number of virtual nodes within constant delay *τ* also increases cross-correlations, since the delays between virtual nodes become shorter. The complexity of a DCR can only be controlled by understanding the tight interplay between the number of virtual nodes and their location, the total delay, the mask structure, and the nonlinearity responsible of mixing past and current inputs [8].

Furthermore, the error margin was exceeded between the desired and target feedback signals in some longitudinal simulations (as in Fig 6). The feedback signal does approach the desired feedback value shortly after a cue. However, it directly starts drifting towards the second desired feedback value within a relatively short timespan of about 2000 reservoir time steps, which is much higher than the limits of fading memory. This drift may be due to suboptimal choice of reservoir parameters or to the mask structure, which may result in insufficient fDCR effective dimensionality to support both stable storage of cue signals and fading memory of input. No parameter optimization was carried out here, since the main goal of demonstrating the potentials of trained feedback was met. Parameter optimization methods are currently under development, and a technique for improving mask structure through plasticity is now available [8]. These tools could provide the way to circumvent the above issue of feedback signal drift.

The current results are only based on numerical simulations, while a rigorous proof of the universal computational power of fDCR remains to be shown. Following the same line of proof as in ODE based reservoirs [25] is not feasible, since sufficient analytical tools to deal with nonlinear delay differential equations are still unavailable [36]. A direct benefit to such analytical tools is providing a theoretical basis to the generalizability of the current findings to other nonlinearities. This, in its turn, is highly relevant to successful physical realizations of fDCRs with naturally occurring nonlinearities [33].

Finally, it is tempting to relate delay-based computational architectures such as the DCR to computational biology, especially that delays are abundant in nature. One noted similarity is that both the DCR and single neurons function on multiple time scales. A neuron receives, at different delays, hundreds of signals in the form of postsynaptic potentials (PSPs) from its afferents and integrates these subthreshold PSPs nonlinearly as action potentials emitted at a slower time scale. Similarly, the DCR nonlinearly integrates faster time scale activity of its virtual nodes to generate its output at a slower time scale. The correspondence, however, is not one-to-one, since the DCR, following a high level of activity, does not undergo a reset of its output. Given the current choice of saturating nonlinearity, the DCR acts more as a mean-field [37] or a firing rate model [38], rather than as a single spiking neuron, and DCRs with resetting nonlinearities similar to spiking neurons remain to be tested. In addition, computations at even slower time scales, corresponding to neural networks can be envisaged in a DCR setting by adding extra delay lines [39, 40] or coupling multiple DCRs to one another. In summary, the DCR architecture provides a fertile ground for studying neural computations based on delays, the harvest of which will occupy research for years to come.

## Supporting Information

S1 Fig The readout training procedure avoids overfitting the target. (A) Comparison between desired (blue) and observed (orange) fDCR output signal *d*_3_ in Experiment 3 (zoomed-in, different run from Fig 5). The training procedure results in a readout that is both robust against outliers (*y* < 1) and is capable of tracking the desired target accurately. (B) Scatter plots of the target verses observed output for both training (yellow) and validation (brown) data sets. (C) Correlation coefficient between desired and observed fDCR output for both training (brown) and validation (yellow).
(TIF)

S2 FigComputing the product of random input and ramping feedback signal.(A) Comparison between desired (blue) and observed (orange) fDCR output signal $d\left(\bar{t}\right)=d_{f}^{\text{tf}}\cdot \left|u^{\text{arb}}\left(\bar{t}\right)\right|$. (B) Scatter plots of the target verses observed output for both training (yellow) and validation (brown) data sets. (C) Correlation coefficient between desired and observed fDCR output for both training (brown) and validation (yellow).(TIF)
