# Supplementary figures and images for: Persistent Memory in Single Node Delay-Coupled Reservoir Computing
Source: PLoS One. 2016 Oct 26;11(10):e0165170. doi: 10.1371/journal.pone.0165170 (PMC5081200; doi:10.1371/journal.pone.0165170)

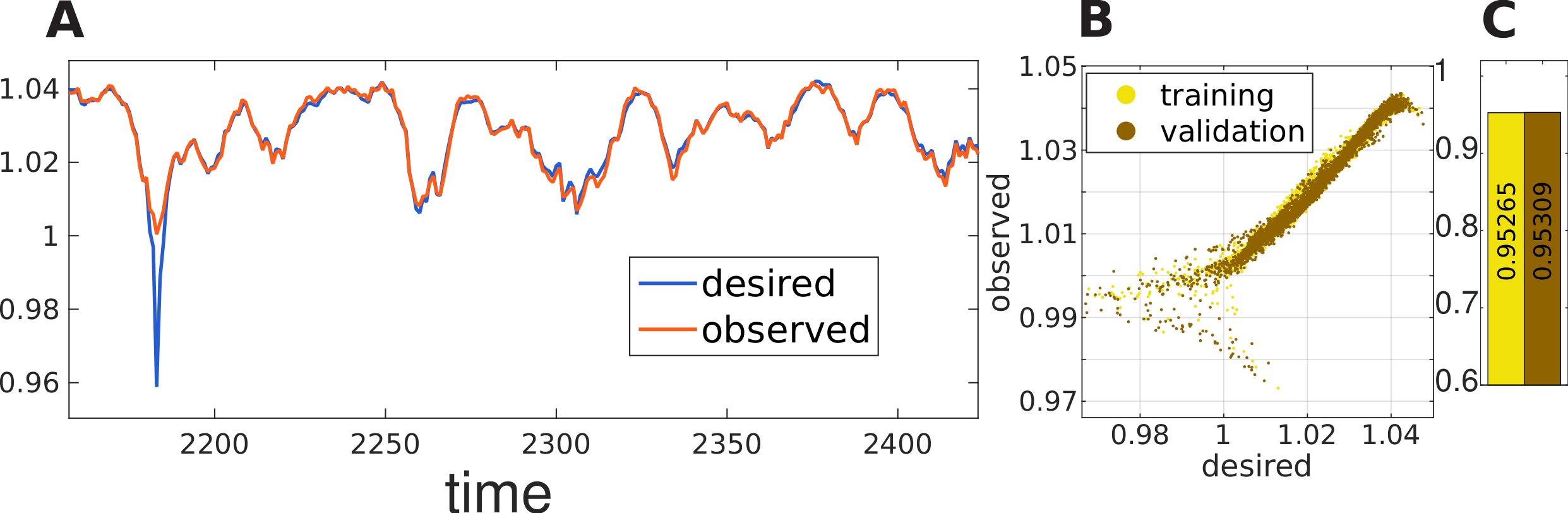

Supplement: S1 Fig — (A) Comparison between desired (blue) and observed (orange) fDCR output signal d3 in Experiment 3 (zoomed-in, different run from Fig 5). The training procedure results in a readout that is both robust against outliers (y < 1) and is capable of tracking the desired target accurately. (B) Scatter plots of the target verses observed output for both training (yellow) and validation (brown) data sets. (C) Correlation coefficient between desired and observed fDCR output for both training (brown) and validation (yellow). (TIF) [file pone.0165170.s001.tif]

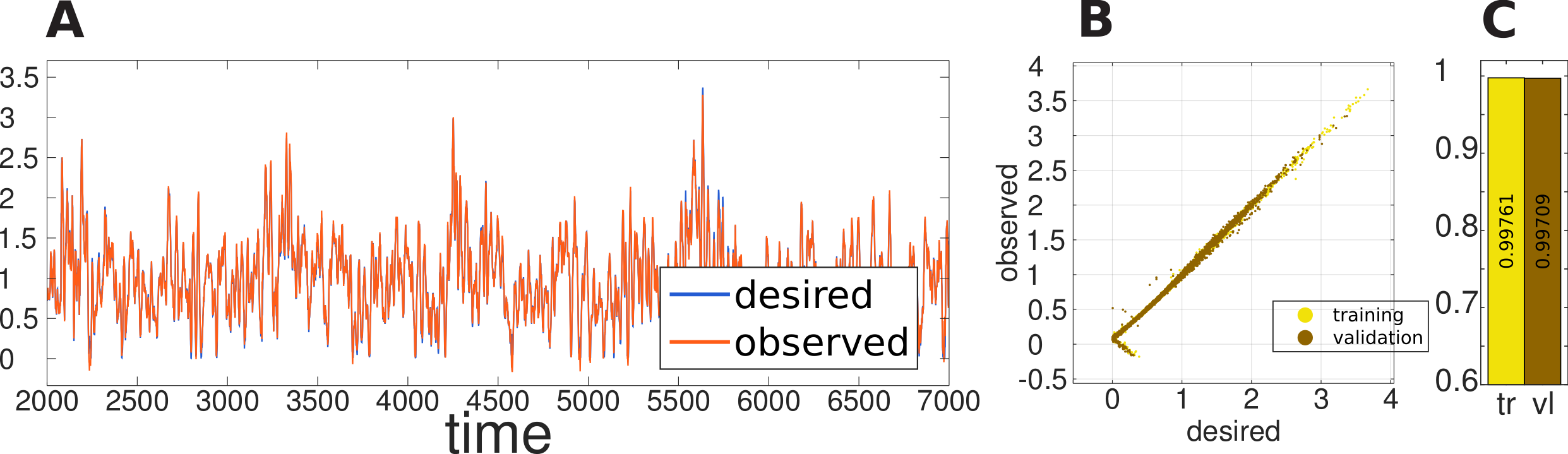

Supplement: S2 Fig — (A) Comparison between desired (blue) and observed (orange) fDCR output signal d(t¯)=dftf·|uarb(t¯)|. (B) Scatter plots of the target verses observed output for both training (yellow) and validation (brown) data sets. (C) Correlation coefficient between desired and observed fDCR output for both training (brown) and validation (yellow). (TIF) [file pone.0165170.s002.tif]
